# A novel index-based decision support toolkit for safe reopening following a generalized lockdown in low and middle-income countries

**DOI:** 10.1038/s41598-021-93415-1

**Published:** 2021-07-08

**Authors:** Abu S. Shonchoy, Khandker S. Ishtiaq, Sajedul Talukder, Nasar U. Ahmed, Rajiv Chowdhury

**Affiliations:** 1 Department of Economics, Florida International University, Miami, FL 33199 USA; 2 Institute of Environment, Florida International University, Miami, FL 33199 USA; 3 Department of Computer Science, School of Computing, Southern Illinois University, Carbondale, IL 62901 USA; 4 Department of Epidemiology, Robert Stempel College of Public Health & Social Work, Florida International University, Miami, FL 33199 USA; 5 The Centre for Non-Communicable Disease Research (CNCR), Monipuripara, Tejgaon, Dhaka, 1215 Bangladesh

**Keywords:** Diseases, Infectious diseases, Viral infection, Health care economics, Health care, Health policy

## Abstract

While the effectiveness of lockdowns to reduce Coronavirus Disease-2019 (COVID-19) transmission is well established, uncertainties remain on the lifting principles of these restrictive interventions. World Health Organization recommends case positive rate of 5% or lower as a threshold for safe reopening. However, inadequate testing capacity limits the applicability of this recommendation, especially in the low-income and middle-income countries (LMICs). To develop a practical reopening strategy for LMICs, in this study, we first identify the optimal timing of safe reopening by exploring accessible epidemiological data of 24 countries during the initial COVID-19 surge. We find that a safe opening can occur two weeks after the crossover of daily infection and recovery rates while maintaining a negative trend in daily new cases. Epidemiologic SIRM model-based example simulation supports our findings. Finally, we develop an easily interpretable large-scale reopening (LSR) index, which is an evidence-based toolkit—to guide/inform reopening decision for LMICs.

## Introduction

The Coronavirus Disease 2019 (COVID-19), caused by severe acute respiratory syndrome coronavirus-2 (SARS-CoV-2), continues to spread worldwide^1,2^.This global health crisis killed millions and infected many folds. It also has a significant economic cost, with output contractions predicted across the vast majority of low-income and middle-income countries (LMIC), and lasting detrimental impacts on fundamental determinants of long-term economic growth prospects in these resource-constrained settings^3,4^. In line with this, it now appears that countries which have the highest COVID-19 death rates, are also among those which suffered the most severe economic downturns^5^. Eventually, given a lower economic resilience and an ever-growing socio-political pressure on the national governments, strict nationwide social distancing interventions (i.e., general lockdown), therefore, had to be lifted either entirely, or in part, by gradually opening selected sectors like hospitality and education.

In many LMICs, large-scale reopening was, however, done abruptly, and in some instances, amidst a continually rising disease count. This premature easing of the community lockdown was followed by adopting various individual (e.g., hygiene practices, facial mask provisions, and physical distancing) and health system measures (e.g., test-trace-isolation of symptomatic cases and their contacts)^6^. However, further inefficiencies (e.g., poor adherence to individual measures, insufficient testing and contact-tracing, low rate of self-isolation among those who were “traced”) increased the likelihood of new generalized outbreaks in these resource-poor countries. In this regard, subsequent waves have since emerged in Europe^7^ and elsewhere, where the resurgence was dealt with new lockdowns^8,9^.

In a national or global epidemic scenario, lockdowns have conventionally shown to be an effective measure in reducing the contact rates within the population; and thereby, in lowering onward transmission^10^. However, important strategic uncertainty remains on the lifting principle of these restrictive measures. While most countries worldwide imposed the strict lockdowns to tackle the initial waves of COVID-19, they differed significantly on the timing of lifting these interventions and reopened while on varying stages of the epidemic trajectory^11^. This variation reflects, at least in part, a lack of relevant literature, since the available decision support tools typically focus on public health strategy prioritization (e.g., by forecasting contagion risk under different intervention scenarios)^6,12,13^ rather than informing the timing of lifting the strategies. This is of particular relevance to the LMICs where complex, resource-intensive approaches to monitor the epidemic growth in real time by generating effective reproduction number or *R* estimates remain unfeasible. In this regard, World Health Organization (WHO) recommends case positive rate of 5% or lower, continuing for two weeks, as a single global threshold to inform safe reopening^14^, However, this generalized approach lacks flexibility to account for context-specific social, structural and economic diversities, especially across the LMIC. Against this backdrop, there remains an urgent need for a standardized evidence-driven approach, which could be utilized as a guiding tool for prompt economic reopening (while reducing the likelihood of a rapid resurgence), in the current and future pandemics.

To address this uncertainty, we have conducted a comprehensive study that aims to: (1) characterize the timing pattern of successful reopening by analyzing global epidemiological data from 24 countries during the initial wave of COVID-19; (2) assess the socio-economic and structural determinants of successful reopening; and (3) develop and validate a simple, evidence-based toolkit to support the reopening decision following a general or localized lockdown in diverse global settings.

## Methods

### Data collection

We systematically collected the COVID-19 epidemiological data from the *Worldometer* electronic database^1^, which sources high-frequency data from respective government official websites of relevant ministries/institutions and also from these authorities' official social media accounts for all countries that had reported a nationwide lockdown between March 1 to April 15, 2020 (i.e., the “first wave” of COVID-19 outbreak in most countries). We further validated the *Worldometer* estimates, where appropriate, by comparing with two additional public data sources: “Our World in Data” by Oxford University and COVID-19 data tracker of the John Hopkins University. We selected countries which: (1) had necessary COVID-19 epidemiologic data available from February 1 to June 3, 2020; (2) had reported at least 500 cases; and (3) were either within 95.5 percentile of the overall mortality or incidence per million population estimates. The collected epidemiological data includes daily number of cases, recoveries, deaths and tests from 24 countries representing a considerable geographical and economic gradient. We also assembled the country-specific lockdown and economic reopening dates based on exhaustive search from different online sources. Further, we have collected Social Progress Index (SPI), Human Development Index (HDI), Worldwide Governance Indicators (WGIs), gross domestic product (GDP) per capita, and google mobility reduction data for each of the study countries to identify potential attributors of successful reopening. Further, details on the data collection sources are available in the Supplementary Material.

### Identification and evaluation of countries with successful reopening

To identify and evaluate each of the study countries based on their reopening characteristics, we employed longitudinal time-series analyses by plotting 5-day moving average of daily infection and recovery estimates from each country. We defined a country as “successful” if following the lifting of the lockdown, the observed daily recovery estimates remained higher than the daily new cases for a continuous period of 30 days. Using these pre-specified criteria, 16 countries out of 24 were found to be “successful”. These countries represented all continents and all income categories (9 belonging to the high-income countries, HICs; and 7 were LMICs)^15^.

### Determination of the key dimensions of successful reopening

We conducted Pearson correlation analysis and multivariate factor analyses (FA) with *varimax* orthogonal rotation in order to linearly characterize the determinants of reopening decision in the included countries. FA is a statistical method that utilizes the linear relationships among continuous variables under study to reduce them into smaller clusters of summary “factors”, retaining as much variance in the original variables as possible. In this analysis, we considered a broad range of potential determinants of successful reopening: gross domestic product (GDP) per capita, “mobility reduction” as a proxy of lockdown execution (calculated as the average percent decline in retail, entertainment, and workplace mobility from the baseline data, obtained from Google Mobility Reports)^16^, human development index (HDI, a statistic composite index of life expectancy, education and per capita income indicators, which are used to rank countries into four tiers of human development)^17^, social progress index (SPI, measures the extent to which countries provide for the social and environmental needs of their citizens)^18^, and worldwide governance indicators (WGI, which includes accountability, political stability, government effectiveness, regulatory quality, rule of law and control of corruption indicators)^19^. The goal of applying FA is to identify the major attribute set from the above-mentioned factors that potentially impacted the re-opening decision.

### Development of a large-scale reopening index

We employed the following steps to develop a novel large-scale reopening (LSR) toolkit to help countries to identify the potential reopening date after lockdown. First, we analyzed 5-day moving average estimates for infected (*I*) and recovered (*R*) cases from all successful countries to construct the country-specific *I-R* trajectories over a 3-month period. Second, from each study country, we extracted data on four key daily variables: new deaths per million ($D\left( t \right)$), positive cases per million tests ($P\left( t \right)$), and new cases per million ($C\left( t \right)$). Third, we created corresponding daily sub-indices for *C*, *D*, and *P* variables, where maximum and minimum values are the corresponding estimates for all included countries, by employing Eqs. (1), (2), and (3).

1 $$ Sub - index\;time - series\;of\;C,~ = ~1 - \left\{ {\frac{{C\left( t \right) - C\left( {min} \right)}}{{C\left( {max} \right) - C\left( {min} \right)}}} \right\} $$

2 $$ Sub - index\;{\text{ }}time - series\;of\;P,~ = ~1 - \left\{ {\frac{{P\left( t \right) - P\left( {min} \right)}}{{P\left( {max} \right) - P\left( {min} \right)}}} \right\} $$

3 $$ Sub - index\;time - series{\text{ }}of\;D,~ = ~1 - \left\{ {\frac{{D\left( t \right) - D\left( {min} \right)}}{{D\left( {max} \right) - D\left( {min} \right)}}} \right\} $$

here, min and max in the parenthesis indicate minimum and maximum values of corresponding $D\left( t \right)$, $P\left( t \right),$ and $C\left( t \right)$ daily time-series combining 24 case study countries. In these included countries, the maximum value for death per million population was 18·81, cases per million population was 189·07, and positive cases per million tests was 53·42. However, local country specific min–max normalization values could be used, alternatively, to construct the sub-indexes.

Fourth, we applied a multiplier (*m*) that denotes the deficit between daily recovered and new cases as shown in Eq. (4).

4 $$ Daily\;Multiplier,m\left( t \right)~ = \frac{{R\left( t \right)~ - ~I\left( t \right)}}{{R\left( t \right)~ + ~I\left( t \right)}} $$

where $R\left( t \right)$ and $I\left( t \right)$ represent 5-day moving average daily recovery and daily infection (cases) time-series data.

Fifth, we constructed country-specific daily LSR index estimates, using the following equation (Eq. 5):

5 $$ LSR\;Index\;\left( t \right) = m\left( t \right) \times \left\{ {\frac{{I_{C}  + I_{P}  + I_{D} }}{3}} \right\} $$

Note that, in the LSR index, all the sub-indexes are equally and additively weighted, to facilitate index calculation even in the absence of any of the sub-index estimates due to data limitation or unavailability. Better weighting mechanisms, such as geometric mean, would require all the time series sub-indexes available for the entire estimation period to compute the daily LSR index, which would be unrealistic for the resource limited countries.

Finally, to account for any potential variations by socioeconomic and governance factors, we adjusted the indices by a multiplication factor (ω) as follows (Eq. 6):

6 $$ Adjusted\;{\text{ }}LSR\;{\text{ }}Index\;\left( t \right) = \omega  \times LSR\;~Index~\;\left( t \right) $$

where ω is a scaled average of GDP, SPI, HDI, and WGI. To estimate ω, we first normalized (0–1) GDP, SPI, HDI, and WGI to bring all of these variables in a comparable scale as follows (Eq. 7):

7 $$ X_{{n,i}}  = \frac{{X_{i}  - X_{{min}} }}{{X_{{max}}  - X_{{min}} }} $$

here, $X_{{n,i}}$ is the normalized values (denoted by *n*) of each country *i* specific variables (i.e., GDP, SPI, HDI, and WGI), $X_{i}$ is the corresponding value before normalization, $X_{{min}} ~$ and $X_{{max}}$ refer to the minimum and maximum values of the variables. We then estimated ω for each country from the average of the corresponding normalized variables (Eq. 8).

8 $$ \omega _{i}  = \frac{{X_{{n,i}}^{{GDP}}  + X_{{n,i}}^{{SPI}}  + X_{{n,i}}^{{HDI}}  + X_{{n,i}}^{{WGI}} }}{4} $$

Based on LSR estimates and Infection-Recovery (I-R) dynamics, we define “waiting period” (WP) as the number of continuous days, following the *I*-*R* crossover, that a country waited to reopen the economy.

Once we plug-in all the country specific required daily time-series data, the proposed LSR index will produce a daily value with the directionality feature (positive or negative sign) assigned with the index. Here, positive LSR index means higher daily recovery than new cases, while a negative LSR index will indicate the opposite. Continuous negative LSR index observed by any country indicates that the daily infection is much higher than the recovery rate, which may warrant some mobility restrictions or NPI (non-pharmaceutical interventions) measures to control the spread. Similarly, to ease the restrictions, continuous positive LSR index could assist safe reopening timing, aiding governments to make a decision. Supplementary Fig. S3 has a detailed flow-diagram explaining the LSR Index construction and decision making for the low- and middle-income countries.

### Example modeling to simulate the impact of index-driven opening

To demonstrate the impacts of actual versus index-driven reopening, we conducted mathematical modelling based on the epidemiologic Susceptible-Infected-Recovered-Mortality (SIRM) compartmental model^20^. Under this framework, we first fitted the observed number of case-data for countries of interest and then executed a hypothetical simulation to investigate the impacts of 'premature' versus ‘safe’ reopening scenarios on the likelihood of triggering a second infection wave and extended lockdown days.

We employed the following four conventional equations (Eqs. 9–12) of the SIRM model:

9 $$ \frac{{dS}}{{dt}} =  - \beta SI $$

10 $$ \frac{{dI}}{{dt}} = \beta SI - \gamma I - \mu I $$

11 $$ \frac{{dR}}{{dt}} = \gamma I $$

12 $$ \frac{{dM}}{{dt}} = \mu I $$

In these equations, *S, I*, *R and M* denote susceptible, infected, recovered and mortality, respectively; while parameters β, $\gamma$, and µ represent transmission, recovery and mortality rates, respectively. For Germany and Iran (selected as two example countries to illustrate the successful and unsuccessful scenarios, respectively), various clinical parameters and transmission dynamics used for the SIRM analyses have been summarized in Supplementary Table S1. Briefly, to fit the observed infection (*I*) curve, we assumed initial susceptible/exposed (*S*_o_) estimates of 24,000 and 12,500, and basic reproduction number (*R*_o_) of 2·8 and 2·6, respectively (which are within the best-estimated *R*_*o*_ range for COVID-19)^21,22^. Then, to simulate the impacts of reopening in the SIRM model, we amplified the number of exposures at the reopening date by 50%, as a proxy for time varying effective reproduction number (*R*_t_), for illustrative purpose, to generate a hypothetical second wave. For this resurgence curve simulation, we used the actual country-specific reopening timing and then compared that with the index-based (desired) reopening.

## Results

### Key characteristics of the included countries

Various demographic, socioeconomic, structural and lockdown-related characteristics of the 24 included countries have been summarized in Table 1. Given large variability in the WP among countries and small sample countries studied, we prefer to use median estimates, which is more stable and do not get influenced by one or two extreme values. Our estimates show that the median WP for HIC is 26 and LMIC is 15 days. The WP in the majority successful HICs and LMICs were longer than 14 days, whereas only two countries (Italy and Malaysia) had a WP of less than 7 days. By contrast, all unsuccessful countries had a WP shorter than 7 days (median duration was 0 days) (Table 1). Additionally, compared with the unsuccessful countries, all successful HIC and LMICs had generally higher average HDI, SPI and WGI estimates (Table 1). However, the average reduction in the overall mobility estimates during lockdown were broadly similar across all included countries. Saudi Arabia was the only HIC among all unsuccessful countries.

**Table 1:** Lockdown, reopening, demographic, and economic characteristics of the 24 countries included in the study.

Country	Lockdown and reopening characteristics				Demographic and economic characteristics				
	Date of lockdown	Date for reopening	Date for *I-R* CROSSOVER	Waiting period, days	GDP/capita, in '000 USD	Mobility Reduction, %	HDI, 2018	SPI, 2019	WGI, 2018
**Successful high income countries**									
Australia	4/2/2020	5/14/2020	4/5/2020	39	49.38	− 33.31	0.94	88.02	1.58
Germany	3/21/2020	5/6/2020	4/14/2020	22	52.56	− 45.13	0.94	88.84	1.50
New Zealand	3/25/2020	5/13/2020	4/10/2020	33	40.75	− 63.30	0.92	88.93	1.81
Denmark	3/3/2020	5/10/2020	4/13/2020	27	54.36	− 33.45	0.93	90.09	1.68
Austria	3/16/2020	4/30/2020	4/4/2020	26	53.88	− 68.04	0.91	86.40	1.46
Spain	3/14/2020	5/26/2020	4/25/2020	31	39.04	− 75.82	0.89	87.47	0.81
Italy	3/9/2020	5/4/2020	4/29/2020	5	40.92	− 70.21	0.88	85.69	0.49
Japan	4/7/2020	5/24/2020	4/30/2020	24	42.07	− 23.06	0.92	88.34	1.34
Singapore	4/8/2020	6/2/2020	5/13/2020	20	94.11	− 62.15	0.94	83.23	1.64
Overall, mean (SD)	–	–	–	25.2 (9.6)	51.89 (16.90)	− 52.72 (19.24)	0.92 (0.02)	87.45 (2.07)	1.37 (0.44)
Overall, median				26					
**Successful middle/low income countries**									
Turkey	3/21/2020	5/4/2020	4/25/2020	9	28.00	− 47.91	0.81	67.49	− 0.48
Croatia	3/23/2020	5/11/2020	4/18/2020	23	14.95	− 34.02	0.84	79.21	0.46
Mali	3/25/2020	7/24/2020	7/9/2020	15	0.93	− 12.09	0.43	45.98	− 0.96
Malaysia	3/18/2020	6/10/2020	6/8/2020	2	11.14	− 39.05	0.80	74.17	0.43
Thailand	3/21/2020	6/15/2020	4/10/2020	66	7.79	− 30.05	0.77	67.47	− 0.20
Vietnam	4/1/2020	4/23/2020	4/5/2020	18	2.74	− 32.87	0.69	68.85	− 0.33
Hungary	3/27/2020	5/18/2020	5/7/2020	11	28.80	− 41.68	0.85	78.77	0.46
Overall, mean (SD)	–	–	–	20.6 (21.1)	13.48 (11.24)	− 33.95 (11.37)	0.74 (0.15)	68.85 (11.26)	− 0.09 (0.56)
Overall, median				15					
**Unsuccessful countries**									
Romania	3/25/2020	5/12/2020	5/10/2020	2	26.66	− 51.34	0.82	74.81	0.16
Saudi Arabia	3/15/2020	5/31/2020	5/26/2020	5	53.89	− 44.95	0.86	63.95	− 0.23
Iran	3/13/2020	4/11/2020	4/9/2020	2	20.89	–	0.80	65.15	− 1.00
Pakistan	3/23/2020	4/10/2020	NA	0	5.54	− 53.60	0.56	48.20	− 0.97
Colombia	3/25/2020	4/27/2020	NA	0	14.50	− 62.96	0.76	70.31	− 0.18
Poland	3/31/2020	4/20/2020	NA	0	29.92	− 33.93	0.87	81.25	0.65
Ghana	3/30/2020	4/23/2020	NA	0	4.50	− 45.00	0.60	61.75	0.05
Ukraine	3/16/2020	5/11/2020	NA	0	8.70	− 43.73	0.75	66.97	− 0.68
Overall, mean (SD)	–	–	–	1.1 (1.8)	20.6 (16.6)	− 47.93 (9.14)	0.75 (0.12)	66.55 (9.76)	− 0.28 (0.58)
Overall, Median				0					

Mobility reduction during lockdown is calculated from the Google Mobility reports for each country.

I-R crossover denotes time point when the daily recovery rate exceeds the daily new case rate.

*I-R* infection-recovery; *GDP* gross domestic product; *HDI* human development index; *SPI* social progress index; *WGI* world governance indicator; *SD* standard deviation; *NA* not applicable.

### Social and structural determinants of economic reopening

Results of the multivariate-adjusted factor analysis (FA), to investigate any potential clustering of variables associated with successful reopening, have been summarized in Table 2. Briefly, FA indicates that two factors were sufficient to optimally explain the data variance based on the Eigenvalue criterion. Together, they accounted for 57.4% of the variance in the included variables. WGI, GDP, SPI, HDI loaded highly (0.86 to 0.97) on factor 1 where WP loaded moderately (0.47)—indicating that WP (as a proxy indicator for reopening decision) is importantly associated with governance, socio-economic, and human development vectors. In contrast, there was weak loading of WP on factor 2 (0.06), indicating a weak representation of WP in this factor. In both factors, low to moderate loadings of mobility reduction and lockdown duration (− 0.26 to − 0.01) implied somewhat lesser relevance of these circumstances to WP. Overall, our analysis shows that governance, social-economic, and human development vectors (Factor 1 in Table 2) had higher linear linkages with the WP while the impacts of mobility reduction and lockdown period on WP were relatively less. The country-specific scores derived from the factor analyses have been given in Supplementary Table S2. Additionally, in the subsidiary Pearson’s unadjusted correlation analysis, WGI, GDP, SPI, HDI and lockdown duration were each correlated moderately (r = 0.32 to 0.45) with WP (Supplementary Fig. S1).

**Table 2:** Major extracted factors and associated loadings of waiting period and the potential drivers.

Variables	Factor 1	Factor 2
WGI	0.95*	0.06
GDP per capita	0.97*	0.24
SPI	0.91*	0.41
HDI	0.86*	0.30
Mobility reduction	− 0.01	− 0.16
Lockdown duration	− 0.01	− 0.26
Waiting period	0.47	0.06

*WGI* world governance indicator; *GDP* gross domestic product; *SPI* social progress index; *HDI* human development index. WP was set to zero for the unsuccessful countries.

*Denotes dominant loadings for each factor.

### Features of the proposed large-scale reopening index

**Figure 1:**
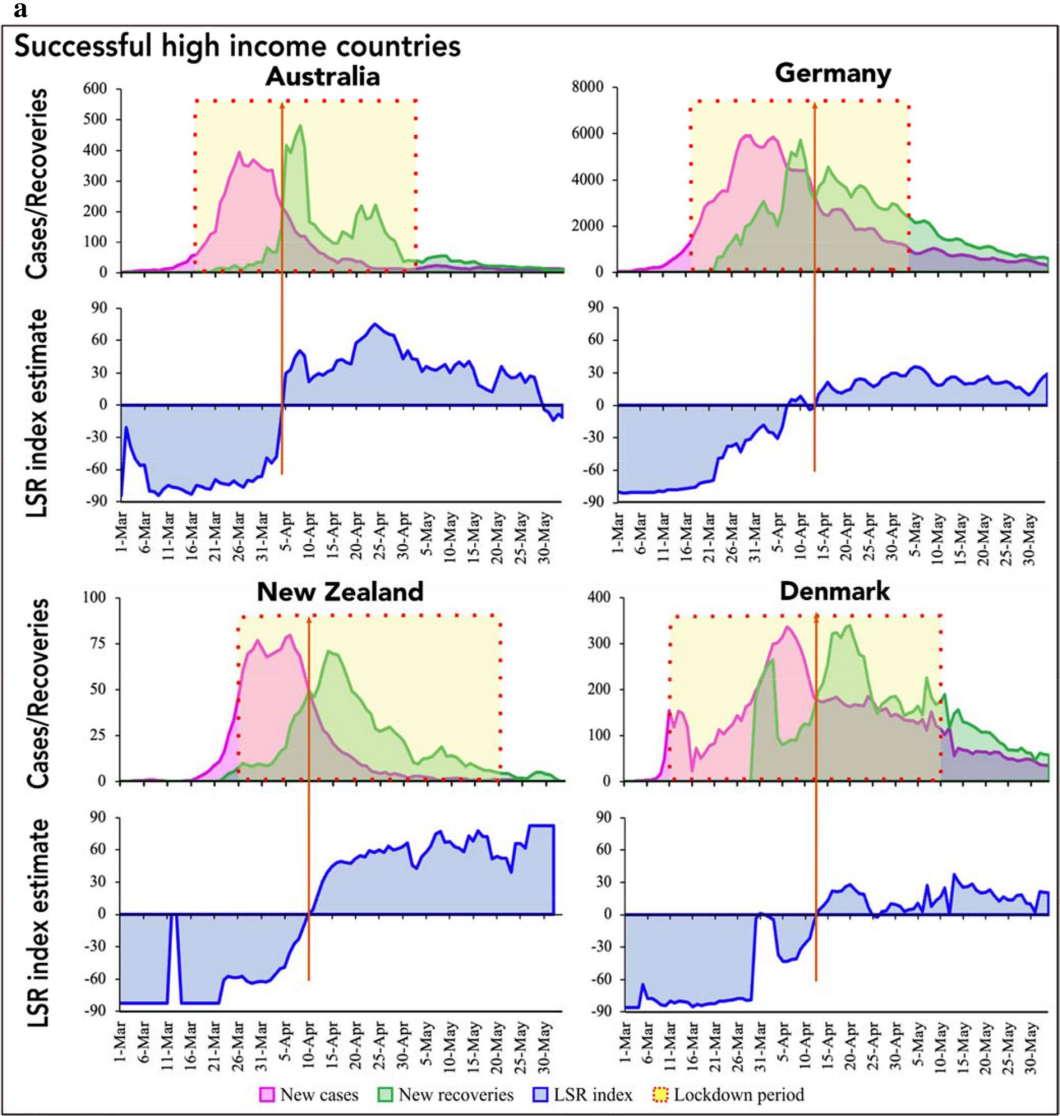

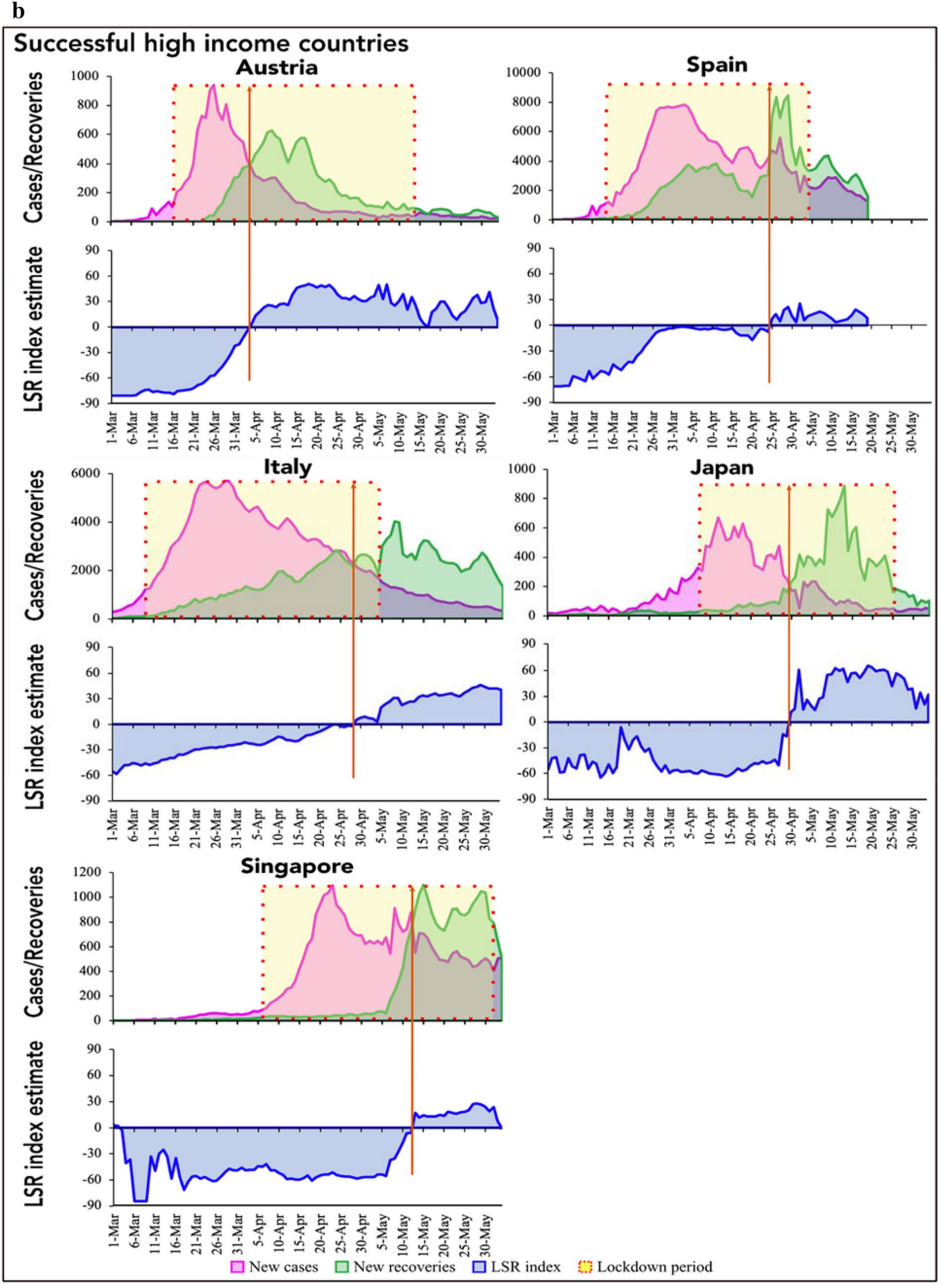
(**a**) Infection-recovery (top panel) and the corresponding LSR index (bottom panel) trajectories in the successful high-income countries (Part a). (**b**) Infection-recovery (top panel) and the corresponding LSR index (bottom panel) trajectories in the successful high-income countries (Part b). The brown vertical line indicates the day of infection-recovery crossover.

**Figure 2:**
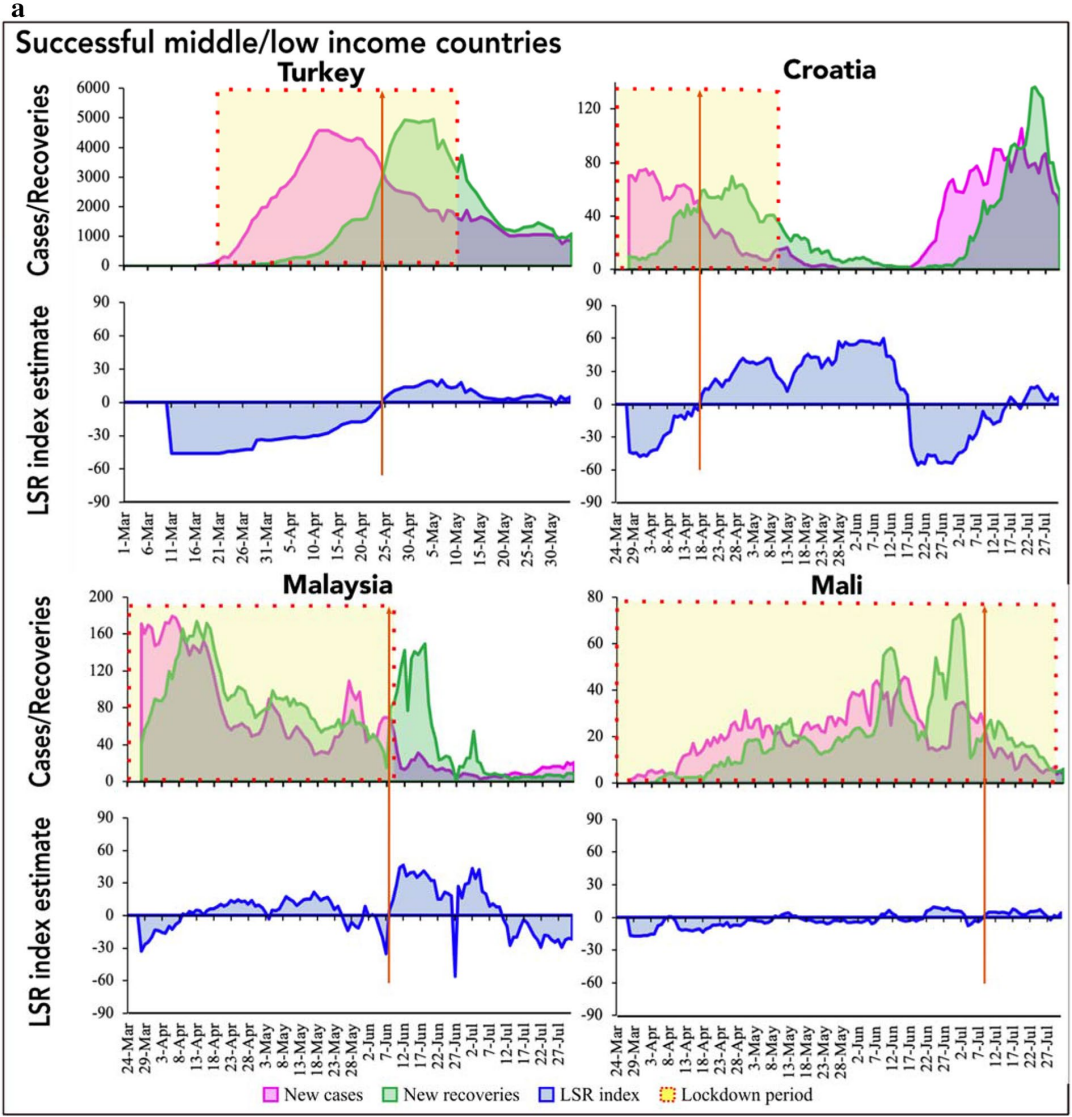

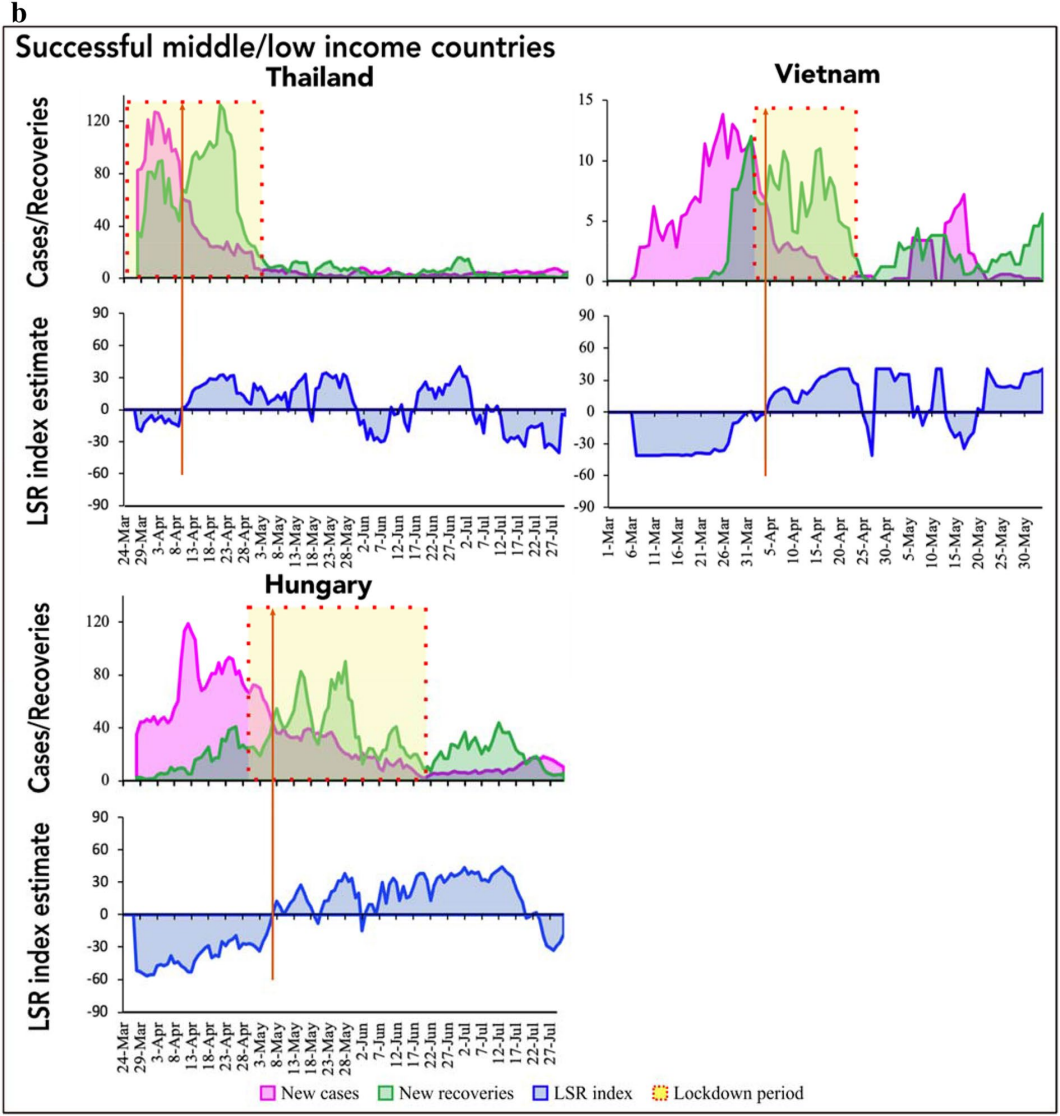
(**a**) Infection-recovery (top panel) and the corresponding LSR index (bottom panel) trajectories in the successful low/middle-income countries (Part a). (**b**) Infection-recovery (top panel) and the corresponding LSR index (bottom panel) trajectories in the successful low/middle-income countries (Part b). The brown vertical line indicates the day of infection-recovery crossover.

Figures 1a,b and 2a,b present *I-R* trajectories (top panel; based on time series COVID-19 data of daily new cases and recoveries), and corresponding LSR index (bottom panel; based on relative slope of infection and recovery from the crossover to reopening) in successful HICs and LMICs, respectively. The corresponding trajectories for the unsuccessful countries have been provided in Supplementary Fig. S2. In all countries, the calculated LSR index, adjusted for country-specific socioeconomic and governance factors (Table 3), aligned well with the country-specific observed *I-R* dynamics, with a neutral value representing the time point of *I-R* crossover and a positive slope denoting that the recovery rates have surpassed the new infection rates.

**Table 3:** Adjusted LSR index by country-specific socioeconomic and governance indicators.

Country	Lockdown duration, days	Crude LSR index at the reopening date	GDP per capita (in '000 USD, scaled)	HDI, 2019 (scaled)	SPI, 2020 (scaled)	WGI, 2018 (scaled)	Average scaling for GDP, HDI, SPI, and WGI	LSR Index at the reopening date (Adjusted)
**Successful high-income countries**								
Australia	42	48.67	0.84	1.00	0.98	0.92	0.93	45.46
Germany	46	43.58	0.71	1.00	0.96	0.89	0.89	38.76
New Zealand	49	70.18	0.64	0.96	0.99	1.00	0.90	63.06
Denmark	68	19.30	0.92	0.98	1.00	0.95	0.96	18.59
Austria	45	40.42	0.77	0.95	0.94	0.88	0.88	35.72
Spain	73	16.00	0.45	0.91	0.92	0.64	0.73	11.69
Italy	56	4.53	0.50	0.89	0.89	0.53	0.70	3.19
Japan	47	74.61	0.61	0.95	0.96	0.83	0.84	62.54
Singapore	55	9.30	1.00	0.99	0.85	0.94	0.94	8.78
Overall, mean (SD)	53.44 (10.74)	36.29 (25.69)	0.71 (0.18)	0.96 (0.04)	0.94 (0.05)	0.84 (0.16)	0.87 (0.09)	31.98 (22.64)
**Successful middle/low income countries**								
Turkey	44	41.19	0.13	0.74	0.46	0.19	0.38	15.57
Croatia	49	37.53	0.22	0.80	0.77	0.52	0.58	21.64
Mali	121	34.81	0.00	0.00	0.00	0.02	0.00	0.15
Malaysia	84	50.93	0.16	0.74	0.65	0.51	0.52	26.27
Thailand	86	85.86	0.11	0.66	0.51	0.29	0.39	33.59
Vietnam	22	69.23	0.03	0.52	0.47	0.24	0.31	21.71
Hungary	52	11.00	0.24	0.82	0.75	0.52	0.58	6.40
Overall, mean (SD)	65.43 (33.30)	47.22 (24.45)	0.13 (0.09)	0.61 (0.29)	0.52 (0.26)	0.33 (0.20)	0.39 (0.20)	17.90 (11.53)
Median waiting period for High LSR (LSR > 20) = 24								
Median waiting period for Low LSR (LSR ≤ 20) = 15								
**Unsuccessful countries**								
Romania	48	13.40	0.19	0.76	0.69	0.41	0.51	6.85
Saudi Arabia	77	21.07	0.35	0.84	0.38	0.27	0.46	9.70
Iran	29	25.08	0.07	0.72	0.44	0.00	0.31	7.73
Pakistan	18	− 46.09	0.01	0.26	0.02	0.01	0.08	− 3.46
Colombia	33	− 52.40	0.09	0.65	0.59	0.29	0.40	− 21.18
Poland	20	− 48.14	0.23	0.87	0.82	0.59	0.63	− 30.16
Ghana	24	− 73.90	0.02	0.33	0.38	0.37	0.28	− 20.35
Ukraine	56	− 26.44	0.04	0.63	0.57	0.11	0.34	− 9.00
Overall, mean (SD)	38.13 (20.60)	− 23.43 (38.19)	0.12 (0.12)	0.63 (0.22)	0.49 (0.24)	0.26 (0.20)	0.38 (0.17)	− 7.48 (15.20)

*LSR* large-scale reopening; *HDI* Human Development Index; *SPI* Social Progress Index; *WGI* World Governance Indicator; *SD* standard deviation.

**Figure 3:**
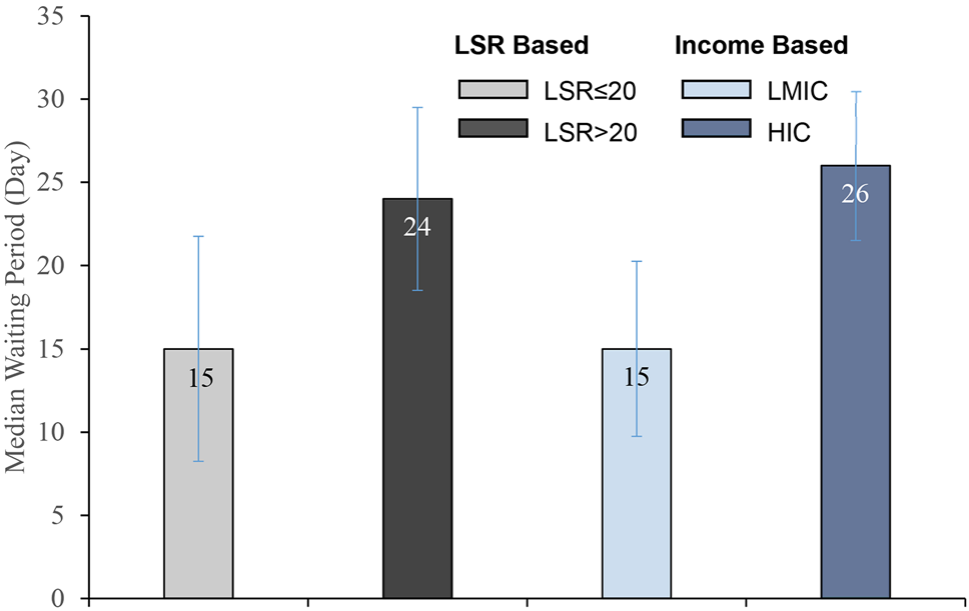
Median of waiting periods by two successful country groups (HIC and LMIC) and LSR index classes (LSR > 20 and LSR ≤ 20). The vertical error bars represent inter-quartile range. *LSR* large-scale reopening; *LMIC* low-income and middle-income countries; *HIC* high income countries.

The adjusted LSR index values differed importantly across successful countries, with the median LSR index value of 35.72 in the HICs and 21.64 in the LMICs (Table 3). We further examined the WP values by two subcategories of adjusted LSR index: (i) a “high positive” index (defined as LSR index > 20), which denotes a higher (and desirable) deviation between recoveries and infections; and (ii) a “low positive” index (defined as LSR index of ≤ 20). We found that for both HICs and LMICs that reopened successfully with the “high positive” LSR index (and therefore had a lower likelihood of immediate resurgence) the median of WP was 24 days, which was 15 days for “low positive” LSR index. (Table 3 and Fig. 3). These LSR based estimates are robustly consistent with our earlier findings with income-based classification of countries, suggesting an approximately minimum 2 weeks WP is required, after the I-R crossover for safe reopening.

### Illustration of the application of LSR index based on SIRM simulation

**Figure 4:**
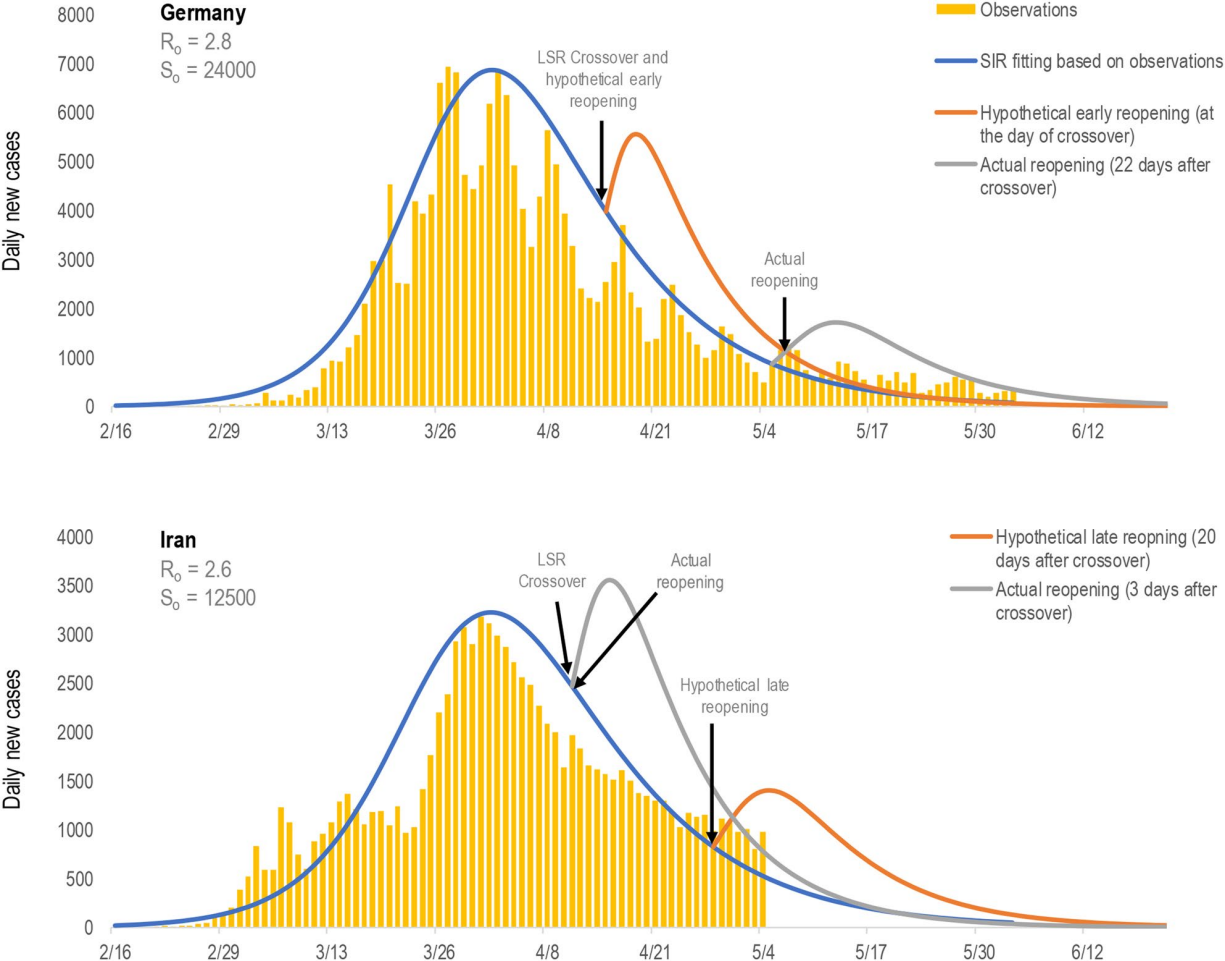
Impact of actual versus hypothetical index-based reopening on new daily cases.

Figure 4 illustrates the likely impact of premature and timely reopening in Germany and Iran, respectively, as examples to illustrate successful and unsuccessful scenarios. The results from the SIRM modelling demonstrate that if Germany had a hypothetical early reopening—e.g., on the day of *I-R* crossover—this would have triggered a significantly higher immediate resurgence. To quantify this difference: there would have been 3,828 additional daily new cases during the simulated “after peak” if reopening happened at the crossover date, compared to the case counts during the peak following the actual reopening date (Fig. 4, top panel).

In contrast, the actual reopening by Iran (i.e., 3 days following the *I-R* crossover) led to a significant resurgence (shown by the orange bars for “observed events”; Fig. 4, bottom panel). However, if Iran reopened 20 days after *I-R* crossover (i.e., in accordance to calculated LSR index for Iran), a significantly lower second peak would have resulted. To quantify, the simulated after peak for the actual reopening would have resulted in 3,534 daily cases, whereas following the LSR-based reopening, there would be only 1,401 daily cases during a simulated after peak (Fig. 4, bottom panel). These results indicate that the LSR-based reopening approach for Iran would have prevented a higher resurgence of the disease.

## Discussion

We found that "successful" countries reopened the economy after the daily recovery rate intersects the infection rate from below while observing the continuous negative trend in the daily new cases. While median WP for successful HIC was 26 days, it was 15 days for successful LMIC, which we propose as the minimum WP threshold (after *I-R* crossover) required for safe reopening. This two-week waiting period for LMIC is consistent with the current epidemiological understanding given that the average incubation period of COVID-19 is approximately 5–7 days from exposure to symptoms onset, with the possibility of incubation periods up to 14 days in some cases^23^. When the daily recovery surpasses the daily new infection, it points towards the effectiveness of the measures taken to control the spread of the virus, bringing the effective reproduction number (*R*_*t*_) down from 2·6 to 0·37^24^. Positive LSR index for a minimum consecutive 14 days, as proposed in this study, suggests that it is likely to prevent a prompt infection wave—providing a safe reopening passage for the economies. Since the infection and mortality risks of COVID-19 vary considerably by age (older population face higher risk), the WP gap observed between HIC and LMIC is also expected—given the lower fertility and relative higher older population in the HIC compared to LMIC^25^.

Although WHO suggested that a case positive rate of 5% or lower, lasting for two weeks, can be an indicator for safe reopening^14^, applying a single threshold for all countries is not optimal given the fact that the LMICs vary widely in: (1) the proportions of missing, duplicative or irrelevant data^26,27^, (2) coverage of testing operations, and (3) country-specific socio-demographic, economic and structural circumstances. Moreover, non-random voluntary testing by individuals, who are either symptomatic or exposed, is imprecise, since estimating the true positivity rate requires regular and repeated testing by a representative sample—irrespective of their illness status. By contrast, our simple, locally-adaptable, index-based reopening strategy entails a more holistic approach as the index integrates (1) both infection and recovery dynamics as a function of number of tests performed, and (2) flexibility to standardize the index by incorporating country-specific social and economic disparities.

Overall, in this study, we have developed, validated and illustrated the use of an easily interpretable toolkit for economic reopening that complements the current WHO prescribed safe re-opening strategy. In particular, this toolkit could be adapted for the LMIC where complex, resource-intensive approaches to monitor the epidemic growth (e.g., by generating real-time effective reproduction number or *R* estimates)^21^ to inform decisions, remain unfeasible. We have developed the LSR index by using empirical data from 24 worldwide countries, and adjusted the indices by relevant local socioeconomic and governance factors. Our results indicate that the adjusted index reflect the corresponding dynamics between the infection and recovery counts accurately. Additionally, for the countries included in this study, a highly successful reopening (characterized by LSR index values of > 20 and a sustained positive slope) demonstrated median 3–4 weeks of waiting period following the infection-recovery crossover. This reinforces the fact that in order to avoid a rapid resurgence (and the protecting health systems overflow), suppression strategies need to be sufficiently prolonged to lower the community transmission rates adequately, given information uncertainty (and detection lag) associated with epidemiologic data. Finally, we found that longer waiting period (as a proxy of reopening decision) is importantly associated with governance, socio-economic and human development factors especially social welfare safeguarding countries.

The LSR index described here comprises several features that may confer important advantages as a novel toolkit. First, our extensive analyses involve and complimentary epidemiological, demographic, structural and social mobility datasets, collected from countries located in diverse economic and geographical settings. This increases the validity and generalizability of this index-based toolkit and provides a global relevance to this work. Second, our methods framework was shaped by established approaches employed in other benchmark global toolkits, such as Human Development Index, where statistical composite indices are constructed based on combining multiple tiers of subnational indicators^17^. Third, our index offers a simplicity of the approach with respect to local adaptability and easy interpretability in a resource-constrained context. For example, adapting this toolkit involves: (a) only a few relatively simple calculating steps, (b) routinely collected disease and other information, and (c) no requirements for considering complex health system-wide data often unavailable in resource-poor settings. Furthermore, the ability of the toolkit to illustrate disease and recovery dynamics in positive or negative quantifiable values can be readily interpretable by the local healthcare authorities and policymakers in the LMICs.

Fourth, our index development involves application of pragmatic equations that do not assume availability of all sub-indices. For example, because the multiplier employed (which determines the index crossover) is principally a function of daily incidence and recovery rates, absence of any sub-index (owing to unavailability of relevant local data, such as tests per million infections) would not affect the overall interpretation, producing estimates broadly similar in direction and magnitude if these data were available. Fifth, the approach, which we used for construction and adjustment of the reopening index, allows further revision and local contextualization. Therefore, it should enable agile updating of the index as relevant new epidemiological (and other related) data emerge about COVID-19. Finally, a flexible method used to construct the indices also implies that, in addition to national lockdowns, this toolkit could also be: (a) incorporated in localized or regional lockdowns, and (b) usefully adapted for other infectious disease epidemics in these geographical areas beyond COVID-19 pandemic.

Since we derived our indices based on country-specific disease dynamics and other publicly-available sub-index data solely from a selected subset of HICs and LMICs with available information, the findings may have somewhat limited the wider scope of our index contextualization. However, our selected HIC and LMIC subsets represent geographical, economical, and population gradients, and therefore could be considered large scale representative set of countries to infer the results. In countries with available information, the quality of recorded data (for example lockdown duration) with respect to completeness and accuracy of data collection, reporting and analysis may differ importantly between high and low-income settings, which could have biased the estimated index^28^. The derived index represents a national-level arithmetic aggregate that could potentially obscure many disparities within the countries (such as by economic, ethnic and gender groupings)^29,30^. Future studies should further uncover any within-country variation (for instance, by regional demographic composition) and adjust the indices accordingly. Our SIRM analyses to estimate the impact of index-based reopening on subsequent resurgence may have been limited by several underlying transmission parameter assumptions used to construct these hypothetical models^20^. As more countries experience infection waves in coming months, further comprehensive modelling studies will, therefore, be needed to better investigate these effects. However, data available to us were insufficient to explore this issue in detail for all included countries.

Our findings may have some implications. To the best of our knowledge, this is the first study that systematically assessed all successfully reopened countries during the initial wave of the COVID-19 pandemic, and analyzed relevant national-level data in order to develop a simple, scalable toolkit for informing economic reopening. The study has a global relevance since achieving a vaccine-induced herd immunity globally may still require years^31–33^, and the success of mitigation interventions (such as test-trace-isolate) has generally been limited worldwide^24,25^. Therefore, suppression strategies, despite their economic consequences, may remain an unavoidable choice to control significant community resurgences of COVID-19 or other future pandemics^34^. Therefore, our toolkit, based primarily on case and recovery rates, offer a potentially more practicable alternative for the LMICs. However, further context-specific research is warranted to tailor this index by local health system circumstances and strategic priorities. The simulated SIRM model was intended to only demonstrate the impact of reopening for two example countries where we did not vary the reproduction number R with time.

In conclusion, our analyses recommend that safe reopening requires minimum two weeks waiting period, after the crossover of daily infection and recovery rates—coupled with post-crossover continuous negative trend in daily new cases. To facilitate this recommendation, we have developed, validated, and illustrated the use of an easily interpretable index as a toolkit for economic reopening. This simple, flexible toolkit could be readily adapted for low and middle-income countries and utilized as a guiding instrument for a prompt reopening of the economy while reducing the likelihood of a rapid resurgence.

## Supplementary Information

Supplementary Information.

## Data Availability

Data used in this study are described in main text, figures, tables, and Supplemental notes.
