# Supplementary material for: A novel index-based decision support toolkit for safe reopening following a generalized lockdown in low and middle-income countries
Source: Sci Rep. 2021 Jul 8;11:14108. doi: 10.1038/s41598-021-93415-1 (PMC8266851; doi:10.1038/s41598-021-93415-1)
Supplement: Supplementary file 1 — Supplementary Information. [file 41598_2021_93415_MOESM1_ESM.docx]

**A Novel Index-Based Decision Support Toolkit for Safe Reopening Following Generalized Lockdowns in Low and Middle-Income Countries**

**SUPPLEMENTARY MATERIAL**

Abu S. Shonchoy,* PhD, Assistant Professor of Economics, Florida International University, Deuxième Maison (DM) 321 , 11200 SW 8th Street, Miami, Florida 33199, USA, Email: [shonchoy@fiu.edu](mailto:shonchoy@fiu.edu)

Khandker S. Ishtiaq, PhD, Research Associate, Institute of Environment, Florida International University, 11200 SW 8th Street, Miami, Florida 33199, USA, Email: [kishtiaq@fiu.edu](mailto:kishtiaq@fiu.edu)

Sajedul Talukder, PhD, Assistant Professor of Computer Science Engineering, A319, School of Computing, Southern Illinois University, 1230 Lincoln Drive, Carbondale, Illinois 62901, USA, Email: [sajedultalukder.cs@gmail.com](mailto:sajedultalukder.cs@gmail.com)

Nasar U. Ahmed, PhD, Associate Professor of Epidemiology , AHC-5 Room 486, Robert Stempel of College Public Health and Social Work, Florida International University, 11200 SW 8th Street, Miami, Florida 33199, USA, Email: [ahmedn@fiu.edu](mailto:ahmedn@fiu.edu)

Rajiv Chowdhury, PhD, Executive Director, The Centre for Non-Communicable Disease Research (CNCR), House 119, Monipuripara, Tejgaon, Dhaka 1215, Bangladesh, Email: [rajiv@cncr-bangladesh.org](mailto:rajiv@cncr-bangladesh.org)

**APPENDIX:**

**Electronic databases review**

For the study, we collected COVID-19 daily number of cases, recoveries, deaths information from the Worldometer electronic database. Positive cases per million test information is taken from the <https://ourworldindata.org/>.^[[1]](#footnote-1)^ The definition used for estimating these variables are given below (taken from the Worldometer). We have also collected different country-specific population, economic, health, and development indicators such as per capita Gross Domestic Product (GDP), Human Development Index (HDI), Social Progress Index (SPI), and Worldwide Governance Indicators (WGI) from corresponding credible sources mentioned below. Furthermore, we obtained country-wise community mobility data over time during lock from the Google COVID-19 community mobility report. The details of epidemiology variables and data sources used in this paper are given below in this Appendix.

**Epidemiology variable definition**

1. Total Death (TD) = Cumulative number of deaths among detected cases.
2. Daily new deaths (D) = Reported daily count of deaths among detected cases.
3. Total Recovery (TR) = Cumulative number of recoveries among detected cases. Worldometer database used the WHO definition of recovered following the criteria of [symptoms resolve + 2 negative tests within 24 hours] or [symptoms resolve + additional 14 days], but in some countries patients discharged from the hospitals are also counted as "recovered" even if no test is performed.^[[2]](#footnote-2)^
4. Daily new recoveries (R) = Reported daily count of recoveries among detected cases.
5. Total Infected (TI) = Cumulative number of infected cases detected.^[[3]](#footnote-3)^
6. Daily new cases (I): Reported daily count of infected cases using the formula [(TI) - (TD) - (TR)].
7. Population in million (Pop): Total population in a country expressed in millions.
8. New Cases per million (C(t)): I/Pop.
9. New Deaths per million (D(t)): D/Pop.
10. Positive cases per million tests (P(t)): Number of COVID-19 positive case detected out of total number of tests performed daily, divided by (Pop).

**Lockdown and reopening data extraction sources**

1. New Zealand: went to alert level 4 on the March 25th and came back to alert level 2 on May 13 (IMF: Policy Response to COVID)

2. Germany: went on Stay-at-home orders on March 21th (https://ourworldindata.org/). On May 6th, the government announced easing for shops, restaurants and sports facilities, where the exact timeline is decided by the state. (IMF: Policy Response to COVID).

3. Austria: Lockdown and reopening dates are taken from the https://ourworldindata.org/

4. Japan: Government announced lockdown on the April 7th and May 25th stat of emergency was listed for all prefectures (IMF: Policy Response to COVID).

5. Australia: Lockdown and re-opening days are taken from <https://ourworldindata.org/>.

6. Turkey: Lockdown data is taken from <https://ourworldindata.org/> and reopening date from the IMF (IMF: Policy Response to COVID).

7. Spain: Lockdown and re-opening days are taken from <https://ourworldindata.org/>.

8. Singapore: Lockdown data is taken from <https://ourworldindata.org/> and reopening date from the IMF (IMF: Policy Response to COVID).

9. Denmark: Lockdown data is taken from <https://ourworldindata.org/> and reopening date from the IMF (IMF: Policy Response to COVID).

10. Iran: On March 25, Iran announced a partial lockdown and ordered a step-by-step reopening of businesses on April 8 (IMF: Policy Response to COVID).

11. Saudi Arabia: Lockdown and re-opening days are taken from <https://ourworldindata.org/>.

12. Italy: Lockdown data is taken from <https://ourworldindata.org/> and reopening date from the IMF. (IMF: Policy Response to COVID).

13. Romania: Lockdown and re-opening days are taken from <https://ourworldindata.org/>.

14. Hungary: Lockdown and reopening data is taken from <https://ourworldindata.org/>

15. Pakistan: Lockdown date is taken from <https://ourworldindata.org/>, reopening date is taken from the following newspaper: <https://www.usnews.com/news/world/articles/2020-05-07/pakistan-to-lift-lockdown-from-saturday-despite-rising-covid-19-curve>

16. Colombia: Lockdown and reopening date from the IMF. (IMF: Policy Response to COVID).

17. Poland: Lockdown data is taken from <https://ourworldindata.org/> and reopening date from the IMF. (IMF: Policy Response to COVID).

18. Ghana: Lockdown data is taken from <https://ourworldindata.org/> and reopening date from the IMF. (IMF: Policy Response to COVID).

19. Croatia: Lockdown and re-opening days are taken from <https://ourworldindata.org/>.

20. Mali: Lockdown data is taken from <https://ourworldindata.org/> and reopening date from the IMF (IMF: Policy Response to COVID).

21. Malaysia: Lockdown and re-opening days are taken from <https://ourworldindata.org/>.

22. Thailand: Lockdown date is taken from <https://ourworldindata.org/>, reopening date is taken from the following newspaper: <https://www.tatnews.org/2020/06/thailand-announces-fourth-phase-of-relaxing-business-and-activity-restrictions-from-15-june/>

23. Vietnam: Lockdown data is taken from <https://ourworldindata.org/> and reopening date from the IMF. (IMF: Policy Response to COVID).

24. Ukraine: Lockdown data is taken from <https://ourworldindata.org/> and reopening date from the IMF. (IMF: Policy Response to COVID).

**Social Progress Index:** https://www.socialprogress.org/index/global/results

**GDP Per capita and total population:** https:// data.worldbank.org

**Governance Indicators:** https://info.worldbank.org/governance/wgi/

**Google Mobility data:** www.google. com. Average over all the index (except for residential) over the lockdown period.

**Epidemiologic data:** https://www.worldometers.info/coronavirus/

**Human Development Index:** http://hdr.undp.org/en/content/human-development-index-hdi

**Supplementary Table 1.** Transmission dynamics and clinical parameters used in the SIRM analysis

| **Parameter** | **Germany** | **Iran** |
| --- | --- | --- |
| *S0* = *E0* | 24,000 | 12,500 |
| Reproduction Number, *R_0_* | 2.8 | 2.6 |
| Initial Infected at *t* = 0 | 20.0 | 20.0 |
| Initial Recovered at *t* = 0 | 1.0 | 1.0 |
| Initial Mortality at *t* = 0 | 1.0 | 1.0 |
| Rate of recovery, *γ* (or the inversed of the duration of the unwell/sick time) | 0.1 (10 days) | 0.1 (10 days) |
| Mortality rate, *μ* | 0.004 | 0.004 |
| Calibration period to fit the Infection curve | February 15-June 3 | February 15-May 4 |
| Infection curve fitting/calibration efficiency (*Nash Sutcliffe Efficiency*) | 0.79 | 0.75 |
| Assumed % of additional people become exposed (in relation to the remaining number of *S* at the day of reopening) | 50% | 50% |

**Supplementary Table 2**. Country-specific scores derived from the factor analyses

| **Country** | **Factor 1** | **Factor 2** |
| --- | --- | --- |
| Australia | 1.32 | 0.67 |
| Austria | 1.09 | 0.12 |
| Denmark | 1.43 | 0.93 |
| Germany | 1.16 | -0.03 |
| Italy | 0.41 | -0.69 |
| Japan | 1.03 | 0.06 |
| New Zealand | 1.27 | 0.03 |
| Singapore | 1.16 | 0.31 |
| Spain | 0.69 | 0.40 |
| Croatia | 0.15 | -0.13 |
| Hungary | 0.11 | -0.45 |
| Malaysia | -0.10 | -0.33 |
| Mali | -1.83 | 2.67 |
| Thailand | -0.33 | 2.67 |
| Turkey | -0.77 | -0.44 |
| Vietnam | -0.85 | -0.22 |
| Colombia | -0.62 | -1.21 |
| Ghana | -0.97 | -0.62 |
| Iran | -1.04 | -0.49 |
| Pakistan | -1.92 | -0.29 |
| Poland | 0.20 | -1.72 |
| Romania | -0.20 | -0.89 |
| Saudi Arabia | -0.59 | 0.10 |
| Ukraine | -0.80 | -0.44 |

**Supplementary Figure 2a.** Infection-recovery (top panel) and the corresponding LSR index (bottom panel) trajectories in the unsuccessful countries (Part a)


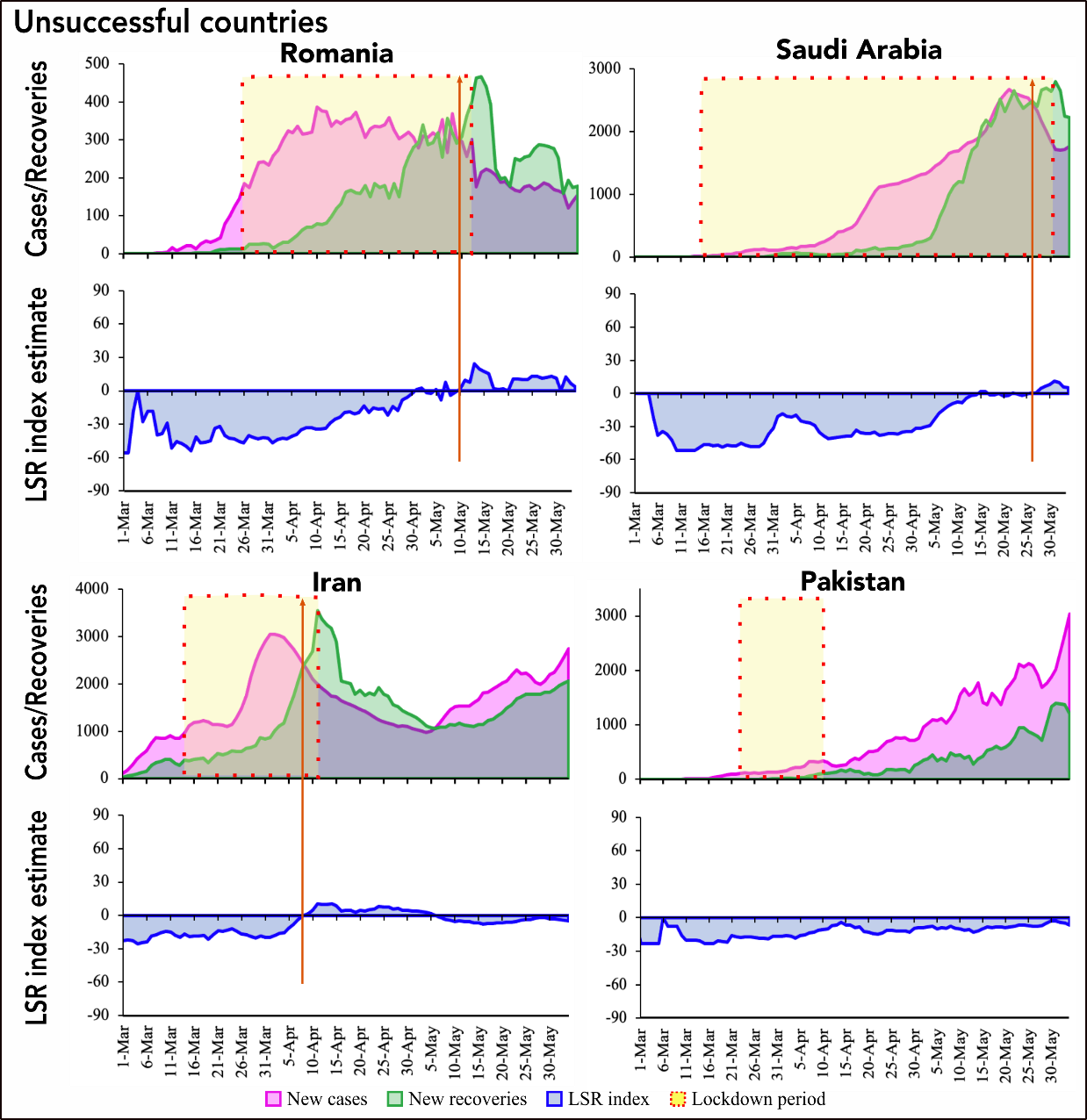


**Supplementary Figure 2b.** Infection-recovery (top panel) and the corresponding LSR index (bottom panel) trajectories in the unsuccessful countries (Part b)


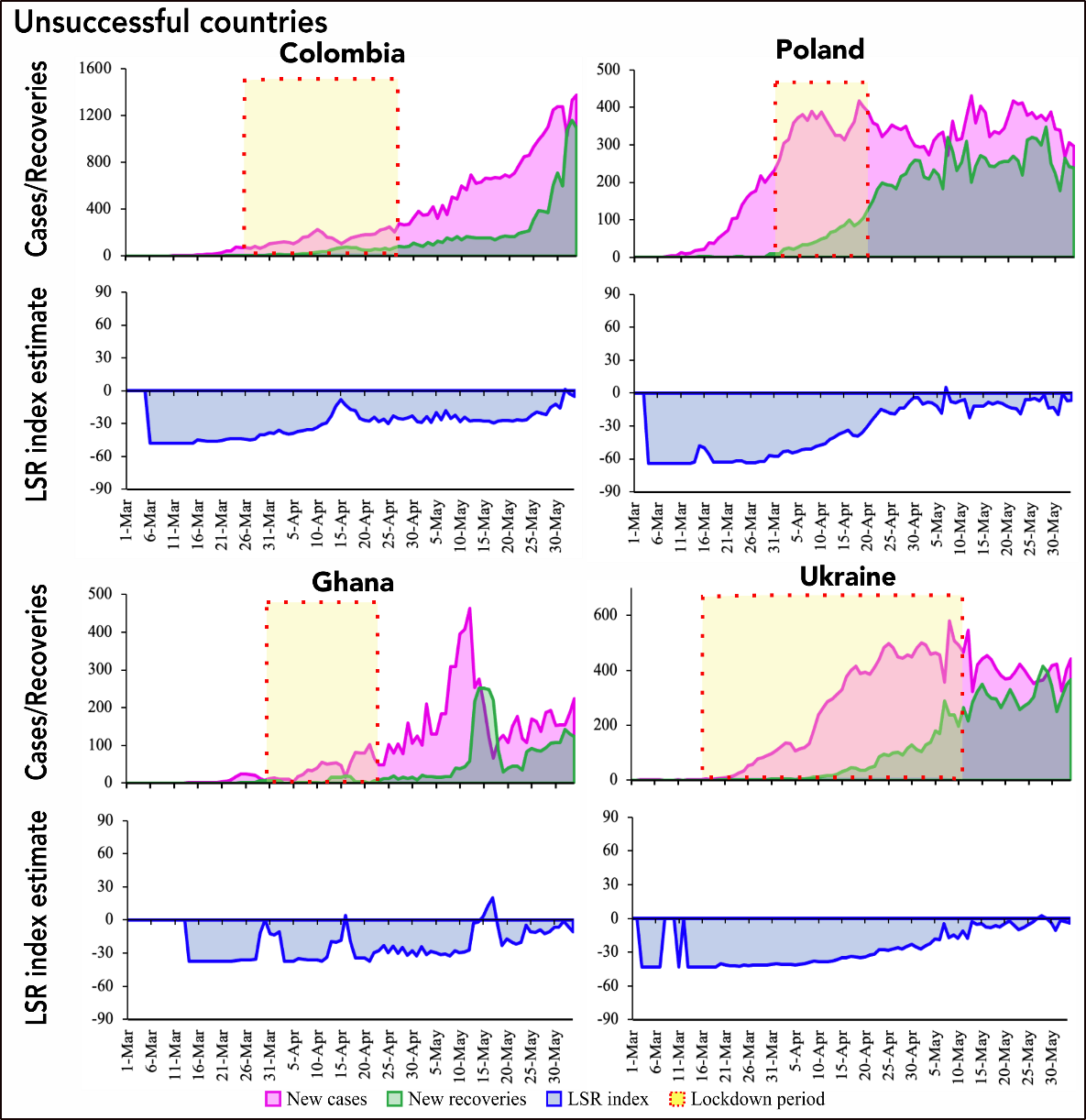


**Figure 3:** LSR Index construction flow-chart

*Note: Inputs marked in Red color is mandatory while inputs marked in Yellow color is optional for developing the LSR Index. If no data available for all the sub-indexes, take the value on 1 and run the LSR index.*

1. Hasell, J., Mathieu, E., Beltekian, D. et al. A cross-country database of COVID-19 testing. Sci Data 7, 345 (2020) [↑](#footnote-ref-1)
2. This statistic is prone to measurement and definition issues, hence should be taken with caution. [↑](#footnote-ref-2)
3. Based on country-specific reporting system this number could capture laboratory and clinically confirmed as well as presumptive, suspect, or probable infected cases. [↑](#footnote-ref-3)
